# Chromosome-Level Genome Assembly of Dybowski’s Frog (*Rana dybowskii*) Provides Insights into Environmental Adaptation and Evolutionary Genomics

**DOI:** 10.3390/ani16132027

**Published:** 2026-07-02

**Authors:** Yuting Liu, Linghao Kong, Jiayu Li, Yingdong Li

**Affiliations:** College of Animal Science and Veterinary Medicine, Shenyang Agricultural University, Dongling Road 120, Shenyang 110866, China

**Keywords:** frog, chromosome-level genome, *Rana dybowskii*, aquaculture genetics

## Abstract

Dybowski’s frog (*Rana dybowskii*) supports a multi-billion-dollar aquaculture sector in northern China and plays a critical ecological role in forest ecosystems. Despite its immense economic value, germplasm degradation and the mystery surrounding its homomorphic sex-determination system present major bottlenecks for the industry. Here, we integrated PacBio HiFi long-read sequencing, Illumina short-read sequencing, and High-Throughput Chromosome Conformation Capture (Hi-C) technologies to assemble the first chromosome-level reference genome of *R. dybowskii*. The final assembled genome size is 3.77 Gb, with a contig N50 of 16.27 Mb and a scaffold N50 of 41.54 Mb. A total of 97.82% of the sequences were successfully anchored onto 12 definitive pseudochromosomes corresponding to haploid chromosome number. Repetitive elements account for 65.61% of the genome, characterized by an unusual dominance of DNA transposons (37.19%) over retrotransposons, suggesting a genomic landscape shaped by extreme cold adaptation. Combining multi-tissue transcriptomic evidence, we structurally predicted 26,862 protein-coding genes, and the predicted gene set showed a BUSCO completeness of 96.1%. Functional annotation successfully categorized 96.55% of the total genes. This genomic resource successfully fills a crucial phylogenetic gap in the *Rana* genus, driving high-efficiency molecular breeding and sustainable conservation of this economic amphibian.

## Simple Summary

Dybowski’s frog (*Rana dybowskii*) is an economically and ecologically vital amphibian in Northeast Asia, primarily farmed for its high-value oviduct (Oviductus Ranae) used in medicine and functional foods. However, sustainable breeding and conservation are severely hindered by a lack of high-quality genomic resources. In this study, we constructed the first chromosome-level reference genome, spanning 3.77 Gb and organized into 12 chromosomes. We annotated 26,862 protein-coding genes and found genomic architecture dominated by DNA transposons, which may link to cold-adaptation mechanisms. This high-resolution genome resolves long-standing industrial bottlenecks by providing an essential molecular platform to accelerate marker-assisted sex control, behavioral traits mapping, and genetic resource conservation in amphibian aquaculture.

## 1. Introduction

Aquaculture is currently the fastest-growing food production sector globally, supplying critical animal protein, essential fatty acids, and micronutrients for the expanding human population [1]. To enhance the sustainability and production efficiency of this sector, genetic improvement of cultured species is essential. Recent advancements in high-throughput sequencing have transitioned aquaculture research into a multi-omics era, where genomics, transcriptomics, proteomics, and metabolomics are increasingly integrated to investigate complex biological traits in economic aquatic animals [2]. These molecular resources have shifted focus from traditional phenotypic selection toward targeted exploration of functional pathways regulating nutrient utilization, disease resistance, and growth endocrinology [3]. Crucially, chromosome-level reference genomes provide the necessary framework to anchor these multi-omics datasets, enabling the precise mapping of economic traits to specific genomic architectures and accelerating marker-assisted or molecular design breeding across diverse aquatic taxa [4].

In several regions, particularly East Asia and parts of Latin America, multiple amphibian species are intensively farmed for food, pharmacological materials, and ecological management [5,6]. The commercial cultivation of various frogs, toads, and giant salamanders has transitioned from traditional, small-scale ranching into structured, large-scale operations that generate substantial biomass and regional economic value [7]. Beyond providing high-quality animal protein, specific economic amphibians produce unique bioactive compounds, polypeptides, and specialized lipids highly utilized in pharmacy and functional food sectors [8]. Consequently, amphibian aquaculture provides critical financial incentives for rural development, establishing itself as a highly distinctive branch of modern aquatic animal production.

Despite their commercial value, amphibians present major genomic challenges due to their large genome sizes, high proportions of repetitive sequences, and extensive transposable elements (TEs) [9,10]. TEs are pivotal drivers of genomic expansion, structural diversification, and regulatory innovation in amphibians [11]. In *Pelophylax*, TE-silencing pathways participate in genome exclusion and clonal inheritance, maintaining genomic stability within hybrid systems [12,13]. Similarly, interspecific hybridization in polyploid Xenopus triggers DNA transposon activation and drives the evolution of piRNA-mediated silencing mechanisms [14,15]. However, while the impacts of TEs on structural evolution and hybridization are increasingly recognized, their functional roles in environmental adaptation and ecological diversification remain poor-ly understood in amphibians. These factors have historically impeded continuous sequence assembly, resulting in fragmented genome drafts. Recently, the integration of long-read sequencing and chromatin interaction mapping has broken these technical bottlenecks, leading to a rapid increase in chromosome-level genome assemblies for various economic frogs [16]. Beyond the classical model organism *Xenopus laevis*, high-quality reference genomes have been established for major commercially farmed species, including the American bullfrog (*Aquarana catesbeiana*) and the spiny-bellied frog (*Quasipaa spinosa*). Furthermore, within the *Rana* genus, chromosome-scale genomic resources have been generated for several ecological and evolutionary representatives, such as *Rana kukunoris* Nikolskii, 1918, *Rana temporaria* Linnaeus, 1758, *Rana chensinensis* David, 1875, and *Rana italica* [17,18]. These foundational datasets have significantly advanced our understanding of amphibian karyotype evolution, genomic organization, and the genetic mechanisms underlying growth, immunity, and environmental adaptation in aquaculture species.

Among economically vital amphibians, the Dybowski’s frog (*Rana dybowskii* Gunther 1876) stands out as a species whose immense commercial success and conservation are heavily bottlenecked by genomic data limitations. Native to Northeast Asia, *R. dybowskii* is highly adapted to cold environments, exhibiting a distinct life cycle characterized by extended hibernation from October to February and breeding from February to June [19,20]. Ecologically, it plays a vital role in controlling forest insect populations [21]. Commercially, *R. dybowskii* supports a large-scale aquaculture sector in northern China, involving over 500,000 practitioners and generating an annual economic output of USD 2.8–3.0 billion [22,23,24]. This industry is primarily driven by Oviductus Ranae (the lipid-rich oviductal secretion of adult females), which commands premium market prices of USD 30 to 50 per kg due to its immunomodulatory, antioxidant, and anti-fatigue properties [25]. However, due to its high market demand and classification as a near-threatened species by the IUCN, wild populations face severe declines and habitat fragmentation from overexploitation. Concurrently, captive farming still relies heavily on wild-caught stocks, leading to germplasm degradation, biosecurity risks, and unregulated translocations [26]. Because females possess virtually all the commercial value, deciphering the species’ evolutionary labile sex-determination system to generate “all-female” cohorts, alongside characterizing genes linked to cold adaptation and behavioral rhythms, is essential for both population conservation and industry sustainability.

To resolve these industrial bottlenecks and decode the genetic mechanisms governing these critical economic and ecological traits, a high-quality reference genome for *R. dybowskii* is an urgent necessity. In this study, we integrated PacBio HiFi long-read sequencing, Illumina short-read sequencing, and high-throughput Chromosome Conformation Capture (Hi-C) to construct the first chromosome-level reference genome assembly for *R. dybowskii*. The final assembled genome spans approximately 3.77 Gb, with 97.82% of the sequences successfully anchored onto 12 pseudochromosomes, corresponding to the haploid chromosome set, yielding a scaffold N50 of 41.54 Mb and a genome assembly BUSCO completeness was 92.0%. A non-redundant reference set of 26,862 protein-coding genes was functionally annotated, revealing that repetitive elements account for 65.61% of the genome. This high-resolution genomic assembly provides a valuable genomic resource for the genus Rana, offering a foundation for future comparative genomic, transcriptomic, and proteomic studies, with potential applications in molecular breeding, genetic management, and conservation of this economically important amphibian.

## 2. Materials and Methods

### 2.1. Ethics Statement

Although *R. dybowskii* is listed as “Near Threatened” on the IUCN Red List, in China it is regarded as an important economic frog species and is neither endangered nor nationally protected. All experiments were performed in accordance with the guidelines for scientific purposes, animal care, and use formulated by the Animal Ethics Committee of Shenyang Agricultural University (protocol code SNLL25101501 and date of approval 20251015). All animal surveys were conducted in compliance with the guidelines established by the Shenyang Agriculture University Experimental Animal Management Committee.

### 2.2. Specimen Collection and Tissue Sampling

On 15 November 2025, 10 male adult *Rana dybowskii* (mean weight 25.18 g ± 7.62 g) were collected from a commercial breeding facility in Dandong, Liaoning Province, China (40°7′30″ N, 124°23′0″ E). The specimens were transported to the Aquatic Biology Laboratory of Shenyang Agricultural University and temporary maintained under standardized laboratory conditions (20–23 °C, 60–70% relative humidity, and a 12 h light/12 h dark cycle).

For whole-genome sequencing, a single healthy adult male was selected. Following euthanasia, high-quality genomic DNA was extracted for Illumina short-read, PacBio HiFi long-read, and Hi-C library construction. To provide transcriptional evidence for gene model annotation, six distinct tissue types were harvested from three adult individuals, including liver, heart, kidney, brain, and muscle from male frogs, alongside oviduct tissue from female frogs. All harvested tissue samples were immediately flash-frozen in liquid nitrogen and stored at −80 °C for DNA extraction and subsequent analysis.

### 2.3. Library Construction and High-Throughput Sequencing

For Illumina short-read sequencing, genomic DNA was extracted from the muscle tissue of the selected adult male individual using a modified CTAB protocol. The purity and concentration of the isolated DNA were assessed via agarose gel electrophoresis, a NanoPhotometer spectrophotometer (Implen, Westlake Village, CA, USA), and a Qubit fluorometer (Life Technologies, Carlsbad, CA, USA). A paired-end genomic library with a target insert size of 350 bp was constructed and sequenced on the Illumina NovaSeq 6000 platform (Illumina, San Diego, CA, USA) in paired-end 150 bp (PE150) mode to generate short reads for genomic survey analysis and subsequent base-level error correction.

For long-read sequencing, genomic DNA from the same specimen was prepared and sequenced on the PacBio Revio platform (Pacific Biosciences, Menlo Park, CA, USA) to generate high-fidelity (HiFi) reads for de novo assembly. To construct the chromosome conformation capture (Hi-C) library, fresh blood and muscle cells were cross-linked with formaldehyde, digested with restriction endonucleases, and enriched for ligated DNA fragments to capture spatial proximity information; the resulting library was sequenced on the Illumina platform in paired-end 150 bp (PE150) mode. Hi-C reads were processed using standard pipelines for read mapping and contact matrix construction. Chromosome scaffolding was performed using Hi-C-based assembly tools, followed by manual inspection and correction of potential misjoins based on interaction heatmaps. Concurrently, total RNA was extracted from the six harvested tissues (brain, muscle, liver, heart, kidney, and oviduct) to construct strand-specific RNA-seq libraries, which were sequenced on the Illumina platform to provide transcriptional evidence for subsequent gene model annotation. The final assembly was validated by intra-chromosomal interaction patterns in Hi-C contact maps. All high-throughput sequencing services were performed by Novogene Co., Ltd. (Tianjin, China).

### 2.4. Genome Size Estimation and Chromosome-Level Assembly

Raw data were quality-filtered using fastp (v1.3.3) to remove adapters and low-quality sequences, and a random subset was screened against the NCBI Nucleotide database using BLAST (v2.13.0+) to detect potential microbial contamination. The genome size, heterozygosity, and repeat content of *R. dybowskii* were computationally estimated via 17-mer frequency analysis using Jellyfish (v1.1.11) and GenomeScope (v2.0). The primary contig-level genome assembly was constructed from PacBio HiFi long reads using hifiasm (v0.21.0-r686), from which organellar (mitochondrial) sequences were identified via BLAST and excluded. To elevate the assembly to chromosome level, clean Hi-C reads were mapped to the primary contigs, followed by scaffolding, ordering, and orientation using the 3D-DNA pipeline and ALLHiC (v0.9.8). The final chromosomal contact matrix was manually reviewed and curated using Juicebox (v2.13.07) to correct misassemblies and define definitive pseudochromosome boundaries.

### 2.5. Repeat Sequence Annotation

A custom transposable element (TE) library was constructed de novo using RepeatModeler (v2.0.3) with default configurations. RepeatMasker (v4.1.2-p1) was subsequently employed to interrogate the assembly against both the custom de novo library and the Repbase database to identify and classify genomic repeats. Candidate repetitive sequences shorter than 100 bp or containing more than 5% ambiguous bases (‘N’) were discarded. The remaining non-redundant TE sequences were clustered using uclust and utilized to hard-mask the genome assembly prior to down-stream structural gene prediction.

### 2.6. Gene Model Structure Prediction and Functional Annotation

Structural annotation of the genome was performed using a comprehensive strategy integrating ab initio prediction, homology-based prediction and RNA-Seq evidence to annotate gene models. For gene predication based on Ab initio methods, Augustus (v3.5) and SNAP (2013-11-29) were used in our automated gene prediction pipeline on the repeat-masked genome. Meanwhile, for the prediction of genes based on similarities to existing sequences, published genome sequences in the NCBI database of three related frogs, including *Rana temporaria*, *Xenopus laevis* and *Lithobates pipiens* were collected. These sequences were then used to annotate the gene models in *R. dybowskii* using TBLASTN (v2.2.26; E-value ≤ 1e^−5^), and then the matching proteins were aligned to the genome for spliced alignments using GeneWise (v2.4.1) to infer gene structures.

Transcriptome reads were assembled using Trinity (v2.8.5). RNA-Seq reads from multiple tissues were aligned to the genome fasta using Hisat2 (v2.2.1). RNA-seq data were used only as transcriptional evidence for gene model prediction and not for expression-based analyses. The resulting alignments were used as input for Stringtie (v2.2.1) for genome-guided transcript assembly. Trinity was used for de novo transcriptome assembly. A non-redundant reference gene set was generated by integrating gene predictions from three methods using EvidenceModeler v1.1.1, incorporating terminal exon support from PASA v2.4.135 for gene structure refinement and masked transposable elements as input for gene prediction. Specific gene families of interest were selected for subsequent manual curation by domain experts.

Functional annotation of genes was conducted using a two-pronged approach. Protein sequences were aligned against the NCBI non-redundant protein (NR) database and the Swiss-prot database utilizing DIAMOND (v2.1.9), ensuring efficient sequence alignment. EggNOG-mapper was used to annotate protein sequences, leveraging the Kyoto Encyclopedia of Genes and Genomes (KEGG), Gene Ontology (GO) terms, and Pfam to provide comprehensive functional insights.

### 2.7. Quality Assessment and Technical Validation

The completeness and structural integrity of the chromosome-level assembly and corresponding gene sets were systematically evaluated using Benchmarking Universal Single-Copy Orthologs (BUSCO, v6.0.0) against the conserved vertebrate lineage database (vertebrata_odb10). To assess base-level consensus accuracy, clean Illumina short reads were mapped back to the definitive pseudochromosomes using BWA-MEM. Alignment constraints, genome coverage profiles, and local sequencing depth distributions were calculated via SAMtools to rigorously validate the continuity and fidelity of the final genomic blueprint.

## 3. Results

### 3.1. Genome Survey and DNA Sequence Quality Control

**Figure 1:**
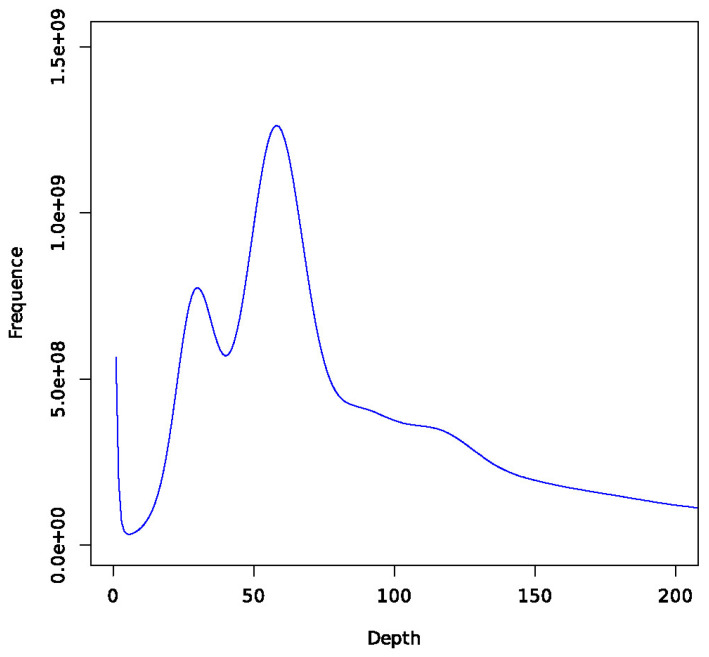
Chromosome-level genome assembly of *Rana dybowskii* K-mer frequency distribution curve.

Illumina NovaSeq 6000 sequencing generated 230.52 Gb of raw paired-end short reads (Table 1). After strict filtration using fastp, the remaining clean data exhibited high base-accuracy, with Q20 and Q30 quality scores reaching 99.19% and 98.38%, respectively, and an average GC content of 44.84%. To evaluate the genomic landscape, a 17-mer frequency analysis was executed using Jellyfish and GenomeScope 2.0, The K-mer distribution displayed a clear bimodal profile (Figure 1). Based on this 17-mer mathematical model, the estimated genome size of *R. dybowskii* was calculated to be 3.31 Gb, with a genomic heterozygosity rate of 1.36% and a repetitive sequence content of 68.98% (Table S1).

### 3.2. Chromosome-Level Genome Assembly and Quality Validation

**Figure 2:**
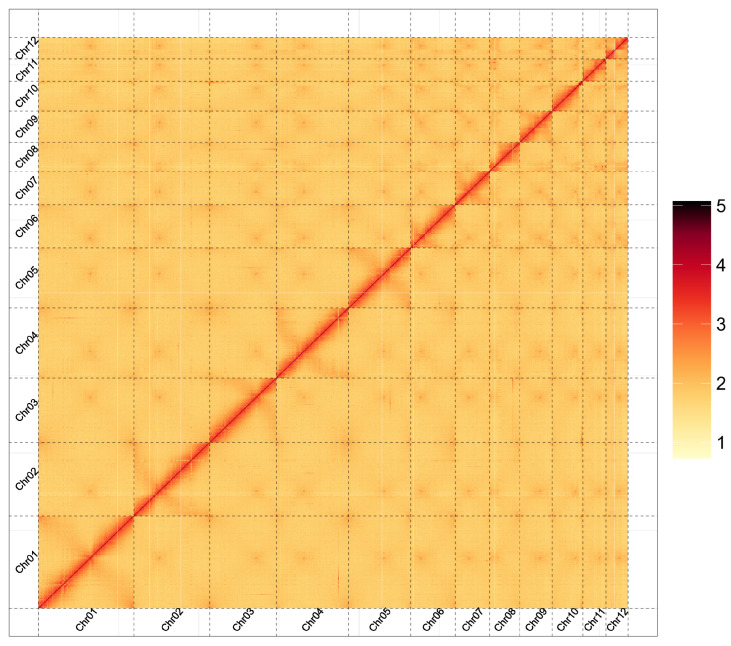
Chromosome-level genome assembly of *Rana dybowskii* Hi-C interaction heatmap, the color bar representing validation of the Hi-C-assisted pseudo-chromosome assembly by calculation of the thermal interaction correlation.

De novo assembly of PacBio HiFi long reads (106.7 Gb) using hifiasm yielded a primary contig assembly of 3.77 Gb, comprising 791 contigs with a contig N50 of 16.27 Mb and a maximum length of 91.51 Mb (Table 1). Integrating 489.4 Gb of Hi-C interaction data via ALLHiC and the 3D-DNA pipeline anchored 3.69 Gb (97.82%) of the contig sequences onto 12 definitive pseudochromosomes (Figure 2). The final assembly exhibited a scaffold N50 of 41.54 Mb and a maximum scaffold length of 501.99 Mb (Table 2). BUSCO analysis of the genome assembly against the vertebrata_odb10 database identified 3086 complete orthologs, indicating a genome assembly completeness of 92.0%, including 3034 single-copy (90.5%) and 52 duplicated (1.5%) genes, with 57 fragmented (1.7%) and 211 missing (6.3%) orthologs (Table 3). Base-level consensus validation using BWA-MEM showed that 98.40% of the clean Illumina short reads mapped back to the assembly, achieving 98.95% genome-wide coverage at an average sequencing depth of 55.55X.

### 3.3. Repetitive Element Characterization

**Figure 3:**
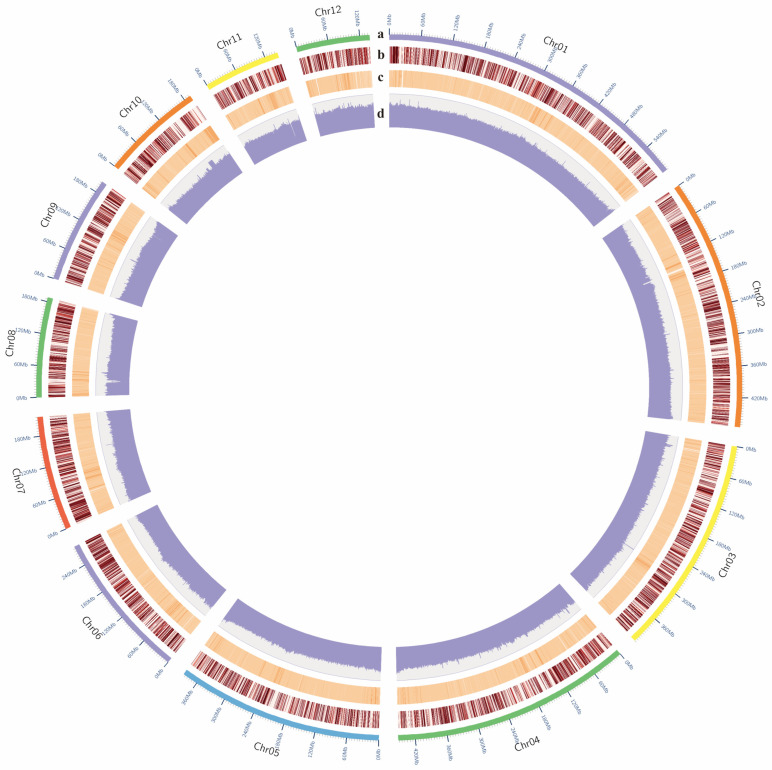
Genomic features of *Rana dybowskii*. Tracks from the outermost to the innermost circle separately represent (**a**) sizes of the 12 pseudochromosomes (Mb), (**b**) Gene density, (**c**) Repeat sequence density, (**d**) GC content.

Repetitive elements spanned 2,473,706,726 bp, accounting for 65.61% of the *R. dybowskii* genome assembly (Table 4). Transposable elements (TEs) constituted the majority of these genomic repeats. Among classified TE categories, DNA transposons were dominant, encompassing 1,403,284,334 bp and representing 37.19% of the total assembly. Retrotransposons comprised the second largest group, covering 1,029,910,240 bp (27.29%). Within the retrotransposon superfamily, Long Terminal Repeat (LTR) elements were the most prominent subclass, spanning 590,443,006 bp (15.65%), followed by Long Interspersed Nuclear Elements (LINEs) at 426,854,233 bp (11.31%) and Short Interspersed Nuclear Elements (SINEs) at 12,613,001 bp (0.33%). The chromosomal distribution and density of these repetitive elements across the 12 pseudochromosomes were visualized in the genomic landscape map (Figure 3).

### 3.4. Gene Model Structure Prediction

The integrated annotation pipeline utilizing ab initio genomic prediction, protein homology mapping, and six-tissue RNA-seq transcripts identified a consensus set of 26,862 protein-coding genes within the *R. dybowskii* genome. BUSCO analysis of the predicted gene set against the vertebrata_odb10 database recovered 3225 complete single-copy orthologs, representing 96.1% completeness. including 3114 single-copy (92.8%) and 111 duplicated (3.3%) genes, with only 49 fragmented (1.5%) and 80 missing (2.4%) orthologs recorded (Table 3).

### 3.5. Functional Annotation of Predicted Genes

**Figure 4:**
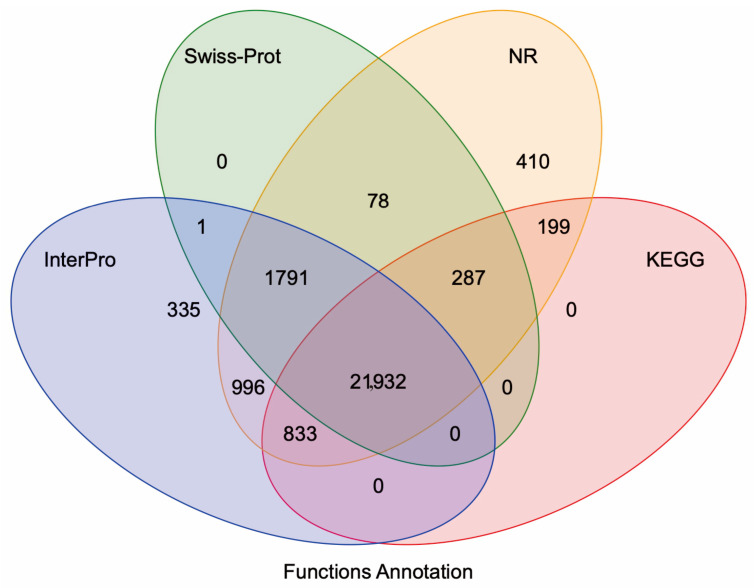
Venn diagram of functional annotation of *Rana dybowskii* genes across databases.

Functional annotation successfully assigned functional terms to 25,934 of the 26,862 predicted genes, representing an overall annotation rate of 96.55% (Table 5). Specifically, 25,610 genes (95.34%) exhibited significant alignments against the NCBI Non-Redundant Protein (NR) database, and 23,257 genes (86.58%) were validated via the UniProtKB/Swiss-Prot database. For pathway and category classifications, eggNOG-mapper assigned functional descriptors to 22,448 genes (83.57%) in the Kyoto Encyclopedia of Genes and Genomes (KEGG) database, 19,996 genes (74.46%) in the Gene Ontology (GO) database, and 22,236 genes (82.78%) in the Pfam database. A multi-database intersection analysis revealed a robust core of 21,932 genes concurrently supported by all four major functional databases, underscores the high reliability of the final annotated genomic blueprint (Figure 4).

## 4. Discussion

The *Rana dybowskii* genome assembled in this study spans 3.77 Gb, exhibiting the characteristic genomic gigantism distinctively common among temperate ranid frogs, while striking a sharp contrast with smaller model anuran genomes such as *Xenopus laevis* (2.1 Gb) [27]. Structurally, the genome size of *R. dybowskii* represents an intermediate evolutionary node within the Eurasian brown frog lineage, being more compact than the high-altitude adapted *Rana kukunoris* (4.83 Gb) and *Rana temporaria* (4.11 Gb), yet larger than its sympatric relative *Rana chensinensis* (3.0 Gb) [18,28,29]. When contrasted with the American bullfrog (*Aquarana catesbeiana*), a premier global aquaculture gigant with a massive estimated genome of approximately 6.37 Gb, *R. dybowskii* showcases a more streamlined yet highly complex macro-chromosomal framework [30]. Backed by a contig N50 of 16.27 Mb and a scaffold N50 of 41.54 Mb, this assembly effectively establishes a high-fidelity reference standard for the genus *Rana*. In comparison, the recently reported *R. chensinensis* genome, despite achieving a higher scaffold N50 of 484.14 Mb due to Hi-C scaffolding, was assembled with a substantially lower contig N50 of only 2.34 Mb. By contrast, the highly contiguous primary assembly of *R. dybowskii* provides a reliable chromosome-scale genomic resource that will facilitate future studies on genome evolution, adaptation, sex determination, and conservation genetics in frogs. Notably, the chromosome-level genome assembled in this study was derived from a single male individual. It does not capture the full spectrum of population-level genomic architecture or structural variations across the species’ geographic range. Future population-scale genomic investigations, incorporating wider geographic sampling and pan-genomic approaches, are warranted to comprehensively assess intraspecific genetic variation, validate transposable element frequencies, and fully elucidate the fine-scale evolutionary dynamics of this species.

### 4.1. Transposable Element Landscapes Driven by Extreme Cold Adaptation

In contrast to the LTR-dominated TE landscapes of *R. kukunoris* (LTRs: 29.65%; DNA: 17.97%) and *R. temporaria* (LTRs: 33.07%; DNA: 22.88%), *R. dybowskii* exhibits a reversed pattern, with DNA transposons (37.19%) far outnumbering LTRs (15.65%) [18,28]. A similar DNA transposon dominance is also observed in *R. chensinensis* (DNA: 36.88%; LTRs: 26.24%) [18]. Geographic overlap among *Pelophylax* species leads to recurrent hybridization [31]. In these hybridogenetic systems, transposable element silencing genes modulate genome elimination and clonal inheritance to maintain genomic stability [32]. The integration of the RanaCR1 retrotransposon into intron 1 of the serum albumin gene in *Pelophylax* frogs illustrates how interspecific contact directly shapes lineage-specific genomic loci [33]. This influx of foreign DNA through asymmetric introgression likely induced a “genomic shock” that disrupted epigenetic silencing, thereby triggering massive DNA-transposon bursts and driving population-level genomic heterogeneity. Unlike LTR retrotransposons suppressed by host defenses, DNA transposons follow distinct post-hybridization trajectories correlated with subgenome-biased DNA loss, restricting further uncontrolled hybridization [14]. In hybridogenesis models, such genomic instability triggers maternal-mediated paternal chromosome loss and genome elimination, resulting in hybrid inviability [12,34]. Intriguingly, *R. dybowskii* primarily inhabits the Changbai Mountain range in northern China, a region sympatrically shared by multiple congeners, including *R. chensinensis*, *R. amurensis* and *Rana huanrenensis* [35]. Notably, divergence-dating and ancestral area reconstructions indicate a Miocene origin for the *R. dybowskii* species complex driven by the orogenic movement of the Changbai Mountain Range, whereas other Northeast Asian anuran lineages diverged later during the Pleistocene [36]. This extended evolutionary timescale and prolonged sympatry provided a vastly expanded temporal window for historical interspecific hybridization and reticulate evolution. Consequently, persistent genetic contact likely destabilized genomic equilibrium, serving as an evolutionary catalyst for the shared DNA-transposon expansion in *R. dybowskii*.

On the other hand, diverse environmental pressures have repeatedly influenced evolutionary trajectories in amphibians. *R. dybowskii*, *R. chensinensis*, and *R. amurensis* share cold-temperate northeastern Asian habitats with prolonged winter freezing. Severe environmental stressors, particularly the extreme low temperatures of high-latitude winters coupled with the profound hypoxia encountered during prolonged aquatic overwintering, can serve as powerful exogenous triggers capable of drastically reshaping transposable element landscapes. The elevational and plateau cold-stress adaptations of *R. kukunoris* [37], as well as the remarkable metabolic reprogramming and molecular resilience to severe oxygen deprivation observed in overwintering *R. amurensis* [38]. This physiological resilience is accompanied by rapid molecular responses. In *R. dybowskii*, low-temperature exposure alters the expression of GLUT4, a key regulator of glucose-mediated cryoprotection, indicating active metabolic adaptation to cold stress [39]. Environmental stress has also been proposed to affect genome evolution through the activation of transposable elements.

We speculate that severe freezing and winter hypoxia in northeastern China act in tandem with historical genomic shocks, triggering localized DNA transposon bursts. Over evolutionary timescales, the adaptive retention of these proliferating elements—potentially harboring and dispersing cis-acting Hypoxia Response Elements (HREs)—could have reconfigured the frog’s hypoxia-responsive networks. This synergetic intersection of historical interspecific hybridization and ongoing extreme climate adaptation provides a robust explanation for the unique DNA-transposon dominance in *R. dybowskii*, offering a crucial genomic foundation for its post-glacial range expansion and active dispersal into harsh sub-frigid niches.

### 4.2. High Genomic Heterozygosity as a Genetic Reservoir for Anti-Stress Selection

The genome of frogs is typically characterized by extensive repeat accumulations [16]. In this study, the heterozygosity rate of *R. dybowskii* was determined to be 1.36%, which reflects the genomic variation in the sequenced individual. This value is notably higher than that of *R. kukunoris* (0.4% to 0.7%), where low heterozygosity is likely a consequence of historical population bottlenecks induced by the harsh, restricted environments of the Tibetan Plateau [28]. Similarly, *R. dybowskii* exhibits superior genetic diversity compared to other economically significant anurans under intensive artificial selection or localized distributions, such as the American bullfrog (*A. catesbeiana*, 0.51%) and the spiny frog (*Quasipaa spinosa*, 0.44%) [30,40]. We hypothesize that the topographically complex terrains of Northeast Asia (specifically across northeastern China, the Russian Far East, and the Korean Peninsula) buffered the species against severe losses of genetic diversity during climatic fluctuations, minimizing genetic drift and preserving substantial standing genetic variation.

From a molecular breeding perspective, maintaining a genomic heterozygosity of 1.36% indicates that the wild population of *R. dybowskii* remains a highly enriched, non-degraded genetic reservoir. This pattern aligns with findings in *Q. spinosa*, where a well-preserved wild genetic diversity (heterozygosity of 0.44%) provides robust raw genetic materials for breeding growth- and disease-resistant traits [40]. In contrast, the critically endangered *Andrias davidianus* has suffered severe inbreeding depression and a dramatic loss of genomic heterozygosity due to a lack of protected wild genetic stocks [41]. This expansive standing variation provides an ideal target pool for identifying molecular signatures related to fitness traits. Breeders can utilize this high-diversity reference to screen for superior wild alleles linked to fast growth, robust immunity, and exceptional overwintering survival, transforming natural evolutionary adaptations into precise markers for molecular-guided genetic improvement [42,43].

### 4.3. Functional Gene Annotation: The Blueprint for Mapping Economic and Behavioral Traits

The multi-source annotation predicted 26,862 protein-coding genes within the *R. dybowskii* genome. This estimate falls comfortably within *R. temporaria* (23,707 genes), while remaining more streamlined than the high-altitude specialist *R. kukunoris* (32,304 genes) [28,29]. The expanded gene repertoire of *R. kukunoris* is largely attributed to the duplication and expansion of specific gene families associated with extreme hypoxia and intense UV-radiation responses on the Tibetan Plateau, an evolutionary pressure distinct from the cold-temperate adaptation of *R. dybowskii* [28]. It aligns closely with the spiny frog (*P. nigromaculatus*, 26,173 genes) but successfully avoids the severe over-prediction or gene-duplication artifacts frequently encountered in the massive genome assembly of the American bullfrog (*A. catesbeiana*, 32,046 genes), where unmasked repetitive pseudogenes often lead to inflated annotations [30,40]. By anchoring our gene models with empirical transcriptomic evidence derived from six distinct tissues, The protein-level BUSCO completeness score was 96.1%, whereas the genome assembly BUSCO score was 92.0%, reflecting BUSCO evaluation at different annotation levels.

In *R. dybowskii* aquaculture, artificial feed adaptation and female ratio improvement are limiting production efficiency. Unlike *A. catesbeiana* and *Q. spinosa*, which have been successfully transitioned to pelleted feeds, *R. dybowskii* typically exhibits a rigid reliance on live prey, making dietary domestication under fully artificial farming conditions exceptionally difficult. The 26,862 protein-coding genes identified in this high-fidelity genome sequence offer an indispensable molecular baseline to explore the genetic architecture governing this behavioral rigidity. In anurans, the transition from movement-triggered striking to stationary feed acceptance relies heavily on a complex interplay between visual perception, olfaction, and central metabolic signaling [44,45]. Functional genomic studies in other frogs highlight that expansions or contractions in specific sensory gene families—such as visual opsins, olfactory receptors, and vomeronasal receptors—directly dictate species-specific foraging niches [46,47]. For instance, while stream-dwelling anurans like *Q. spinosa* rely heavily on expanded chemosensory receptor repertoires for nocturnal foraging, highly visual predators depend on conserved retinal signaling cascades for prey tracking [40]. Furthermore, downstream appetite regulation and feed intake are tightly modulated by central neuroendocrine pathways, specifically the leptin, proopiomelanocortin, and neuropeptide Y networks [48]. Identifying sequence polymorphisms or expression variations in these 26,862 coding models will allow researchers to pinpoint why *R. dybowskii* lacks the behavioral plasticity seen in *A. catesbeiana*. Ultimately, this precise functional inventory shifts the focus from empirical husbandry trials to targeted molecular selection, laying the groundwork for identifying functional markers associated with pelleted-feed tolerance.

### 4.4. A Chromosome-Scale Platform for Sex Determination and Applied Breeding Diagnostics

The exceptional continuity of the *R. dybowskii* genome assembly—highlighted by a remarkably high contig N50 of 16.27 Mb and the successful anchoring of 97.82% of sequences onto 12 definitive pseudochromosomes. The 12 assembled pseudochromosomes perfectly align with the known haploid chromosome number documented in previous karyotype studies of *R. dybowskii* and its close Eurasian congeners like *R. amurensis* [49]. In *R. dybowskii* aquaculture, executing precise sex control is of paramount commercial significance, given that only females yield the economically valuable oviduct (Oviductus Ranae). In evolutionary biology, the genus *Rana* serves as a premier model animal for studying the turnover of homomorphic sex chromosomes and the driving mechanisms behind candidate sex-determining genes, such as Dmrt1, as comprehensively detailed in *R. temporaria* [29]. To date, significant efforts have been made to characterize the molecular dimorphism between sexes [50]; for instance, transcriptomic profiling of *R. dybowskii* during the reproductive period has successfully identified crucial differentially expressed genes (DEGs) between the testis and ovary, offering a fundamental snapshot of gonad-specific regulatory networks [51]. However, without a high-quality physical map, reconciling these expressed transcripts with their macro-syntenic positions on homomorphic sex chromosomes remained a major hurdle.

Crucially, recent investigations suggest that environmental fluctuations can significantly alter the phenotypic sex and secondary sexual characteristics of *R. dybowskii* (unpublished data), revealing a high degree of phenotypic plasticity or potential sex-reversal triggered by external stressors such as thermal anomalies or endocrine-disrupting contaminants. In anurans with homomorphic sex chromosomes, such environmentally induced discordance between genetic sex and phenotypic sex poses a severe threat to sustainable aquaculture, as cryptic mass masculinization can drastically deplete productive female cohorts. By establishing a continuous, near-complete chromosomal coordinate system, this assembly provides an genomic resource genomic map for future population-level resequencing and genome-wide association studies (GWAS) to characterize the cryptic, non-recombining X/Y or Z/W sex-determining regions in *R. dybowskii*. Moreover, as an environmental bioindicator, *R. dybowskii* populations face mounting anthropogenic and environmental stressors [52,53]. Thus, a chromosome-level reference serves as a genomic platform for monitoring environmental pollution and evaluating wild population resilience via fitness-associated locus tracking, providing a genomic resource that may facilitate basic ecological observations of environmental adaptation to future ecological and molecular breeding research.

## 5. Conclusions

In conclusion, this study successfully generated the first high-fidelity, chromosome-level reference genome assembly for *Rana dybowskii* using an integrated multi-omics sequencing approach. The final 3.77 Gb assembly provides exceptionally high continuity (contig N50 of 16.27 Mb) and anchors 97.82% of genomic sequences onto 12 definitive chromosomes. Genomic landscape analyses revealed a high heterozygosity rate (1.36%) representing a non-degraded genetic reservoir, alongside a unique DNA-transposon-dominated repeat architecture potentially driven by long-term adaptation to prolonged winter freezing in Northeast Asia. Furthermore, our robust annotation of 26,862 protein-coding genes provides an accurate coding blueprint that bypasses historical gene-duplication artifacts. This chromosomal dataset provides an indispensable tool for future genome-wide association studies (GWAS) to delineate cryptic sex-determining regions, discover biomarkers for economically vital traits (e.g., oviduct hypertrophy and foraging behavior), and implement precise genetic diagnostics in commercial closed-colony breeding. Ultimately, this study bridges basic evolutionary ecology with applied molecular breeding, ensuring both the commercial sustainability and the wildlife conservation of *R. dybowskii*.

## Figures and Tables

**Table 1:** Library sequencing data and methods used in this study to assemble the *Rana dybowskii* genome.

Sequencing Strategy	Sequence Read Archive (SRA)	Platform	Usage	Clean Data (Gb)	Coverage (X)
Short-read	SRR38417394	Illumina	Genome survey	230.52	68.16
Long-read	SRR38481767	PacBio Revio	HiFi assembly	106.7	31.55
Hi-C	SRR38481764	Illumina	Hi-C assembly	489.4	144.7
RNA-seq of brain	SRR38485805	Illumina	Anno-evidence	6.24	/
RNA-seq of muscle	SRR38485804	Illumina	Anno-evidence	6.41	/
RNA-seq of liver	SRR38485808	Illumina	Anno-evidence	6.9	/
RNA-seq of heart	SRR38485807	Illumina	Anno-evidence	7.21	/
RNA-seq of kidney	SRR38485806	Illumina	Anno-evidence	6.61	/
RNA-seq of oviduct	SRR38485803	Illumina	Anno-evidence	7.33	/

**Table 2:** Statistics of the draft genome.

Total assembly size (Gb)	3.77
Number of contigs	791
Contig N50 (Mb)	16.27
Number of N50	62
Contig N90 (Mb)	2.16
Number of N90	300
Average long (Mb)	4.77
Maximum contig length (Mb)	91.51
Minimum contig length (Mb)	0.17
GC content (%)	43.53
Number of Ns	0

**Table 3:** Statistics of genome assembly.

Type	Summary	Count	Ratio (%)
Genome assembly	Complete BUSCOs (C)	3087	92
	Complete and single-copy BUSCOs (S)	2968	88.5
	Complete and duplicated BUSCOs (D)	119	3.5
	Fragmented BUSCOs (F)	52	1.6
	Missing BUSCOs (M)	215	6.4
	Total BUSCO groups searched	3354	100
Gene annotation	Complete BUSCOs (C)	3223	96.1
	Complete and single-copy BUSCOs (S)	3137	93.5
	Complete and duplicated BUSCOs (D)	86	2.6
	Fragmented BUSCOs (F)	44	1.3
	Missing BUSCOs (M)	87	2.6
	Total BUSCO groups searched	3354	100

**Table 4:** Summary of the repetitive sequences in *Rana dybowskii* genome.

Type	Number	Length (bp)	Percentage (%)
SINE	56,369	7,686,239	0.2
LINE	942,138	335,414,432	8.9
LTR	2,624,759	589,910,914	15.65
DNA	5,951,412	1,402,167,148	37.19
Unknown	405,218	72,831,350	1.93
Total		2,473,706,726	65.61

**Table 5:** Functional annotation of *Rana dybowskii* genes.

Type	Number	Percent (%)
Total	27,823	
NR	26,526	95.34
Swiss-Port	24,089	86.58
KEGG	23,251	83.57
InterPro	25,888	93.05
Pfam	20,917	75.18
GO	20,717	74.46
Annotated	26,862	96.55
Unannotated	961	3.45

Note: (Total refers to all predicted gene models generated by the annotation pipeline, while Annotated refers to genes with functional annotation support or high-confidence filtering).

## Data Availability

The raw reads have been deposited in the NCBI database (BioProject number PRJNA1461158).

## Supplementary Materials

The following supporting information can be downloaded at: https://www.mdpi.com/article/10.3390/ani16132027/s1, Table S1: Statistics of genome survey data.
